# Supplementary material for: How frailty index impacts death in chronic kidney disease: A retrospective observational investigation
Source: PLoS One. 2026 Jan 29;21(1):e0341643. doi: 10.1371/journal.pone.0341643 (PMC12854423; doi:10.1371/journal.pone.0341643)
Supplement: Attachment — Submitted filename: Reviewer Comments for 41253.docx [file pone.0341643.s002.docx]

Reviewer Comments for Manuscript Number PONE-D-25-41253

"How Frailty Index Impacts Death in Chronic Kidney Disease: A Retrospective Observational Investigation"

I. Major Strengths

1.Large-Scale and Generalizable Cohort

The study uses data from the NHANES (1999–2018), a nationally representative U.S. cohort, including 4,350 CKD patients (eGFR < 60 mL/min/1.73m²). This ensures strong external validity—results are not limited to a single center or regional population, but applicable to diverse U.S. CKD patients across age, race, and socioeconomic status.

2.Comprehensive Frailty Assessment

The frailty index (FI) is constructed per Searle’s validated methodology, incorporating 53 deficits across 7 domains (cognition, ADL dependence, depression, comorbidities, healthcare utilization, physical function, laboratory markers). This multidimensional approach avoids limitations of single-domain tools (e.g., gait speed alone) and better captures the complexity of frailty in CKD—where physiological deficits (e.g., anemia, inflammation) and functional impairments (e.g., mobility loss) often coexist.

3.Robust Statistical Analyses

Multivariable adjustment: Three hierarchical Cox models (adjusting for demographics, lifestyle, and comorbidities) minimize confounding, ensuring associations between FI and mortality are not driven by factors like age or diabetes.

Dose-response and stratification: Restricted cubic splines confirm linear associations between FI and mortality (all-cause, cardiovascular, cancer), while subgroup analyses (age, sex, BMI, diabetes/hypertension) validate consistency across populations.

Sensitivity analyses: Excluding patients with preexisting CVD/cancer and multiple imputation for missing data confirm results are robust to selection bias and data gaps.

4.Focus on Cause-Specific Mortality

Most prior CKD-frailty studies focus on all-cause mortality; this study extends knowledge by linking FI to cardiovascular (ICD-10: I00-I09, I11, I13, I20-I51) and cancer (ICD-10: C00-C97) mortality. The finding that high FI increases cancer mortality by 67% (tertile 3 vs. 1) fills a gap in understanding frailty’s role in non-cardiovascular outcomes in CKD.

4. Outcome Interpretation and Reporting

Cancer Mortality Mechanism Underexplained:The study finds FI is associated with cancer mortality (HR=1.67, tertile 3 vs. 1) but does not discuss why—frailty may increase cancer risk via immune dysfunction [28] or delay cancer diagnosis via reduced healthcare utilization.

Suggestion:

In discussion, cite studies linking frailty to cancer (e.g., Reference 22, which shows frailty increases cancer mortality via impaired treatment tolerance) and hypothesize: “In CKD, frailty may exacerbate immune dysfunction (e.g., reduced lymphocyte count, included in FI), increasing cancer progression risk. Future studies should explore FI and cancer treatment outcomes (e.g., chemotherapy adherence) in CKD.”

Kaplan-Meier Curve Reporting Gaps:Figure 2 shows survival curves for FI tertiles but lacks median survival times (e.g., “Median all-cause survival was 8.2 years in FI tertile 1 vs. 4.5 years in tertile 3”). This limits clinical understanding of FI’s impact on survival duration.

Suggestion:

Add median survival times to Figure 2 legends and results (e.g., “Median all-cause survival differed significantly by FI tertile: 8.2 (IQR: 5.1–10.3) years (tertile 1), 6.1 (IQR: 3.8–8.5) years (tertile 2), and 4.5 (IQR: 2.9–6.7) years (tertile 3); log-rank p<0.001”).

II. Key Issues and Revision Suggestions

1. Methodological Gaps in Frailty Index Construction

Over-Reliance on Self-Reported Data: Critical FI components (e.g., comorbidities, ADL dependence, depression via PHQ-9) are self-reported, which may introduce bias (e.g., underreporting of “stigma-related” conditions like depression, or overreporting of functional limitations). Objective measures (e.g., grip strength, 4-meter gait speed—available in NHANES) are not integrated into the FI, despite their validation in CKD frailty assessment [10, 21].

Suggestion:

In the “Frailty Index” section, acknowledge self-report bias and supplement with sensitivity analyses: re-calculate FI by replacing 1–2 self-reported domains (e.g., ADL dependence) with objective NHANES data (e.g., gait speed < 0.8 m/s) to test if associations with mortality persist.

Cite studies validating objective measures in CKD (e.g., Reference 10, which uses grip strength to define frailty) to contextualize limitations of self-report.

No CKD-Specific FI Adaptation: The FI uses general population cutoffs (tertiles: 0.010–0.130, 0.130–0.211, 0.211–0.665) but does not account for CKD-specific physiological deficits (e.g., uremic toxin accumulation, renal anemia, mineral bone disorder). These factors may accelerate frailty in CKD, making general population cutoffs less clinically relevant.

Suggestion:

Add a post-hoc analysis stratifying by CKD stage (G3a: 45–59, G3b: 30–44, G4: 15–29, G5: <15 mL/min/1.73m²) to test if FI-mortality associations vary by renal function. For example, “In CKD G5, the adjusted HR for all-cause mortality in FI tertile 3 was 2.51 (95% CI: 2.03–3.11), stronger than in G3a (HR=1.78, 95% CI: 1.42–2.23).”

Discuss whether CKD stage-specific FI cutoffs (e.g., lower threshold for frailty in G5) may be needed for clinical use.

2. Confounding Factors and Causal Inference

Missing CKD-Specific Confounders:The models adjust for general factors (BMI, hypertension) but omit key CKD-related variables linked to both frailty and mortality:

Urine albumin-to-creatinine ratio (ACR): A marker of renal damage, independently associated with frailty [21] and cardiovascular mortality [3].

Anemia severity: Beyond hemoglobin (included in FI), iron parameters (ferritin, transferrin saturation) influence muscle function and frailty [29].

Inflammation markers: C-reactive protein (CRP) or interleukin-6 (IL-6)—available in NHANES—mediate both CKD progression and frailty [28].

Suggestion:

Add a fourth Cox model (Model 4) adjusting for ACR, ferritin, and CRP. Report if FI associations are attenuated (e.g., “After adding ACR, the adjusted HR for FI tertile 3 decreased from 2.02 to 1.89, suggesting partial mediation by renal damage”).

No Mediation Analysis for Causal Pathways:The discussion hypothesizes mechanisms (inflammation, lifestyle changes) but does not test them. For example, it is unclear if frailty increases mortality via inflammation, or if inflammation independently drives both frailty and mortality.

Suggestion:

Use mediation analysis (e.g., Baron-Kenny method) to test if CRP (inflammation) or physical activity (lifestyle) mediates the FI-mortality association. For example, “CRP mediated 15% of the association between FI and cardiovascular mortality (p<0.001), indicating inflammation is a partial driver.”

III. Overall Recommendation

Revise and Resubmit. The study makes a valuable contribution to CKD and frailty research by linking a multidimensional FI to cause-specific mortality in a large, generalizable cohort. Key revisions—adding CKD stage stratification, including CKD-specific confounders, and enhancing data transparency—will address current gaps and ensure the manuscript meets PLOS ONE’s scientific rigor standards. With these adjustments, the work will provide actionable guidance for integrating frailty assessment into CKD clinical care.
