# Supplementary material for: How frailty index impacts death in chronic kidney disease: A retrospective observational investigation
Source: PLoS One. 2026 Jan 29;21(1):e0341643. doi: 10.1371/journal.pone.0341643 (PMC12854423; doi:10.1371/journal.pone.0341643)
Supplement: Attachment — Submitted filename: Reviewer Comments for 41253R1.docx [file pone.0341643.s004.docx]

Reviewer Comments for Manuscript Number PONE-D-25-41253R1

"How Frailty Index Impacts Death in Chronic Kidney Disease: A Retrospective Observational Investigation"

I. Assessment of Response to Review Comments

The authors have systematically addressed the core issues raised in the initial review, with most key suggestions effectively implemented. The overall quality of revisions is high.

II. Remaining Minor Issues

Inadequate clinical practical details: While the authors suggest integrating frailty management into CKD care, they have not specified clinically actionable intervention thresholds for the frailty index (e.g., whether FI>0.210 constitutes a high-risk threshold for intervention) or screening frequency (e.g., annual screening for CKD G3+ patients). It is recommended to briefly supplement these in the Discussion section to enhance clinical translatability.

III. Core Strengths of the Revised Manuscript

Significantly improved methodological rigor: Added CKD stage-specific subgroup analysis and mediation analysis, adjusted for key confounders such as ACR and CRP, and the statistical model is more aligned with the pathophysiological characteristics of CKD, enhancing the reliability of results.

Convincing conclusions: Using a large, nationally representative cohort (3,262 cases), the study clearly demonstrates a dose-response relationship between the frailty index and all-cause, cardiovascular, and cancer mortality. This association remains robust across subgroups of different CKD stages, sexes, and ages, ensuring strong generalizability.

Comprehensive and objective discussion of limitations: The authors candidly acknowledge data constraints (e.g., lack of treatment information, self-report bias) without avoiding methodological shortcomings, reflecting the scientific rigor of the study.

IV. Overall Review Conclusion

The authors have fully addressed the core issues raised in the initial review. The revised manuscript meets PLOS ONE’s scientific rigor requirements in methodology, statistical analysis, and result interpretation. The core conclusions are reliable and hold significant clinical and public health value. The remaining minor issues do not affect the core quality of the study, and no further major revisions are needed.
